# Bis­(1*H*-benzimidazole-κ*N* ^3^)bis(4-methyl­benzoato-*κ* ^2^ *O*,*O*′)cobalt(II)

**DOI:** 10.1107/S1600536808005722

**Published:** 2008-03-05

**Authors:** Wen-Dong Song, Chang-Sheng Gu, Xiao-Min Hao, Jian-Bin Yan

**Affiliations:** a College of Science, Guang Dong Ocean University, Zhanjiang 524088, People’s Republic of China

## Abstract

In the title mononuclear complex, [Co(C_8_H_7_O_2_)_2_(C_7_H_6_N_2_)_2_], the Co^II^ atom is coordinated by four carboxylate O atoms from two 4-methyl­benzoate ligands and two N atoms from two benzimidazole ligands in an octa­hedral coordination geometry. The molecules are assembled *via* inter­molecular N—H⋯O hydrogen-bonding inter­actions into a three-dimensional network.

## Related literature

For literature on related structures, see: Song *et al.* (2007 ▶).
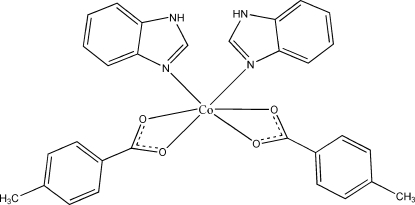


## Experimental

### 

#### Crystal data

[Co(C_8_H_7_O_2_)_2_(C_7_H_6_N_2_)_2_]
*M* *_r_* = 565.48
Monoclinic, 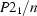

*a* = 13.3209 (4) Å
*b* = 14.5129 (4) Å
*c* = 15.2656 (4) Å
β = 107.020 (1)°
*V* = 2821.97 (14) Å^3^
*Z* = 4
Mo *K*α radiation
μ = 0.65 mm^−1^
*T* = 296 (2) K
0.35 × 0.32 × 0.26 mm

#### Data collection

Bruker APEXII area-detector diffractometer
Absorption correction: multi-scan (*SADABS*; Sheldrick, 1996 ▶) *T*_min_ = 0.805, *T*_max_ = 0.849
36127 measured reflections
6400 independent reflections
4431 reflections with *I* > 2σ(*I*)
*R*_int_ = 0.062

#### Refinement

*R*[*F* ^2^ > 2σ(*F* ^2^)] = 0.050
*wR*(*F* ^2^) = 0.125
*S* = 1.05
6400 reflections
354 parameters
H-atom parameters constrained
Δρ_max_ = 0.60 e Å^−3^
Δρ_min_ = −0.33 e Å^−3^

### 

Data collection: *APEX2* (Bruker, 2004 ▶); cell refinement: *SAINT* (Bruker, 2004 ▶); data reduction: *SAINT*; program(s) used to solve structure: *SHELXS97* (Sheldrick, 2008 ▶); program(s) used to refine structure: *SHELXL97* (Sheldrick, 2008 ▶); molecular graphics: *SHELXTL*-*XP* (Sheldrick, 2008 ▶); software used to prepare material for publication: *SHELXTL*-*XP*.

## Supplementary Material

Crystal structure: contains datablocks I, global. DOI: 10.1107/S1600536808005722/ng2407sup1.cif

Structure factors: contains datablocks I. DOI: 10.1107/S1600536808005722/ng2407Isup2.hkl

Additional supplementary materials:  crystallographic information; 3D view; checkCIF report

## Figures and Tables

**Table 1:** Hydrogen-bond geometry (Å, °)

*D*—H⋯*A*	*D*—H	H⋯*A*	*D*⋯*A*	*D*—H⋯*A*
N2—H2⋯O4^i^	0.86	1.90	2.757 (3)	173
N4—H4*A*⋯O2^ii^	0.86	1.91	2.760 (3)	170

Symmetry codes: (i) 
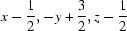
; (ii) 
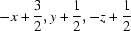
.

**Fig. 1.**
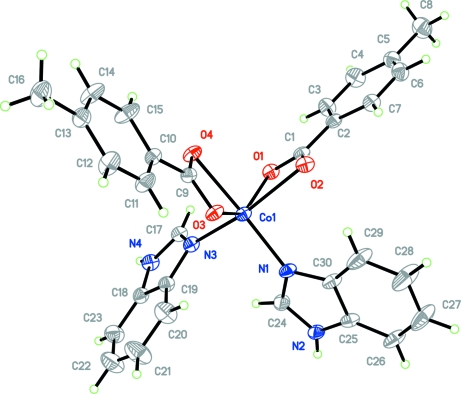
The structure of (I), showing the atomic numbering scheme. Non-H atoms are shown with 30% probability displacement ellipsoids.

**Fig. 2.**
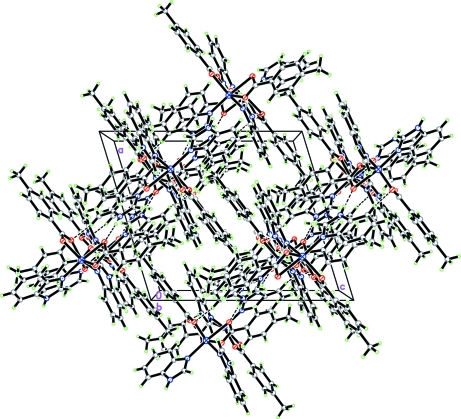
A packing view of the title compound. The intermolecluar hydrogen bonds are shown as dashed lines.

## supplementary crystallographic information

### Comment

In the structural investigation of 4-methylbenzate complexes, it has been found that the 4-methylbenzoic acid functions as a multidentate ligand [Song *et al.* (2007)], with versatile binding and coordination modes. In this paper, we report the crystal structure of the title compound, (I), a new Co complex obtained by the reaction of 4-methylbenzoic acid, benzimidazole and cadmium chloride in alkaline aqueous solution.

As illustrated in Figure 1, the Co^II^ atom exists in a disordered octahedral environment, defined by four carboxyl O atoms from two bisdentate 4-methylbenzate ligands and two N atoms from two benzimidazole ligands. Intermolecular N—H···O hydrogen bonding interactions (Table 1) between the benzimidazole molecules and the carboxyl O atoms of 4-methylbenzate ligands form the structural motif exhibiting non-filled voids. (Fig. 2).

### Experimental

A mixture of cobalt chloride(1 mmol), 4-methylbenzoic acid (1 mmol), benzimidazole(1 mmol), NaOH (1.5 mmol) and H_2_O (12 ml) was placed in a 23 ml Teflon reactor, which was heated to 433 K for three days and then cooled to room temperature at a rate of 10 K h^-1^. The crystals obtained were washed with water and dryed in air.

### Refinement

H atoms were placed at calculated positions and were treated as riding on the parent C atoms with C—H = 0.93 - 0.97 Å, N—H = 0.86 Å, and with *U*_iso_(H) = 1.2 *U*_eq_(C, N).

### Crystal data

**Table:**

[Co(C_8_H_7_O_2_)_2_(C_7_H_6_N_2_)_2_]	*F*_000_ = 1172
*M**_r_* = 565.48	*D*_x_ = 1.331 Mg m^−^^3^
Monoclinic, *P*2_1_/*n*	Mo *K*α radiation λ = 0.71073 Å
Hall symbol: -P 2yn	Cell parameters from 3500 reflections
*a* = 13.3209 (4) Å	θ = 1.4–28.0º
*b* = 14.5129 (4) Å	µ = 0.65 mm^−^^1^
*c* = 15.2656 (4) Å	*T* = 296 (2) K
β = 107.020 (1)º	Block, colorless
*V* = 2821.97 (14) Å^3^	0.35 × 0.32 × 0.26 mm
*Z* = 4	

### Data collection

**Table:**

Bruker APEXII area-detector diffractometer	6400 independent reflections
Radiation source: fine-focus sealed tube	4431 reflections with *I* > 2σ(*I*)
Monochromator: graphite	*R*_int_ = 0.062
*T* = 296(2) K	θ_max_ = 27.5º
φ and ω scan	θ_min_ = 2.0º
Absorption correction: multi-scan(SADABS; Sheldrick, 1996)	*h* = −17→17
*T*_min_ = 0.805, *T*_max_ = 0.849	*k* = −18→18
36127 measured reflections	*l* = −19→19

### Refinement

**Table:**

Refinement on *F*^2^	Secondary atom site location: difference Fourier map
Least-squares matrix: full	Hydrogen site location: inferred from neighbouring sites
*R*[*F*^2^ > 2σ(*F*^2^)] = 0.050	H-atom parameters constrained
*wR*(*F*^2^) = 0.125	*w* = 1/[σ^2^(*F*_o_^2^) + (0.0376*P*)^2^ + 3.4284*P*] where *P* = (*F*_o_^2^ + 2*F*_c_^2^)/3
*S* = 1.05	(Δ/σ)_max_ = 0.001
6400 reflections	Δρ_max_ = 0.60 e Å^−^^3^
354 parameters	Δρ_min_ = −0.33 e Å^−^^3^
Primary atom site location: structure-invariant direct methods	Extinction correction: none

### Special details

**Table:**

Geometry. All e.s.d.'s (except the e.s.d. in the dihedral angle between two l.s. planes) are estimated using the full covariance matrix. The cell e.s.d.'s are taken into account individually in the estimation of e.s.d.'s in distances, angles and torsion angles; correlations between e.s.d.'s in cell parameters are only used when they are defined by crystal symmetry. An approximate (isotropic) treatment of cell e.s.d.'s is used for estimating e.s.d.'s involving l.s. planes.
Refinement. Refinement of *F*^2^ against ALL reflections. The weighted *R*-factor *wR* and goodness of fit *S* are based on *F*^2^, conventional *R*-factors *R* are based on *F*, with *F* set to zero for negative *F*^2^. The threshold expression of *F*^2^ > σ(*F*^2^) is used only for calculating *R*-factors(gt) *etc*. and is not relevant to the choice of reflections for refinement. *R*-factors based on *F*^2^ are statistically about twice as large as those based on *F*, and *R*- factors based on ALL data will be even larger.

### Fractional atomic coordinates and isotropic or equivalent isotropic displacement parameters (Å^2^)

**Table:**

	*x*	*y*	*z*	*U*_iso_*/*U*_eq_	
C1	0.8899 (2)	0.68421 (19)	0.14187 (19)	0.0312 (6)	
C2	0.9812 (2)	0.69664 (19)	0.10609 (19)	0.0330 (6)	
C3	1.0312 (3)	0.7819 (2)	0.1119 (2)	0.0445 (8)	
H3	1.0065	0.8321	0.1373	0.053*	
C4	1.1174 (3)	0.7916 (2)	0.0798 (3)	0.0531 (9)	
H4	1.1504	0.8486	0.0845	0.064*	
C5	1.1560 (3)	0.7186 (2)	0.0409 (2)	0.0443 (8)	
C6	1.1059 (3)	0.6346 (2)	0.0354 (2)	0.0462 (8)	
H6	1.1310	0.5845	0.0102	0.055*	
C7	1.0195 (3)	0.6235 (2)	0.0665 (2)	0.0403 (7)	
H7	0.9864	0.5665	0.0609	0.048*	
C8	1.2497 (3)	0.7308 (3)	0.0059 (3)	0.0640 (11)	
H8A	1.2699	0.6722	−0.0126	0.096*	
H8B	1.3070	0.7561	0.0536	0.096*	
H8C	1.2320	0.7720	−0.0456	0.096*	
C9	0.7811 (2)	0.58438 (18)	0.3444 (2)	0.0301 (6)	
C10	0.7966 (2)	0.52426 (18)	0.42681 (19)	0.0318 (6)	
C11	0.7287 (3)	0.4538 (2)	0.4289 (2)	0.0462 (8)	
H11	0.6711	0.4430	0.3781	0.055*	
C12	0.7449 (3)	0.3984 (2)	0.5059 (2)	0.0538 (10)	
H12	0.6978	0.3511	0.5061	0.065*	
C13	0.8299 (3)	0.4123 (2)	0.5826 (2)	0.0414 (7)	
C14	0.8973 (3)	0.4815 (3)	0.5790 (2)	0.0566 (10)	
H14	0.9557	0.4916	0.6292	0.068*	
C15	0.8818 (3)	0.5371 (2)	0.5031 (2)	0.0543 (10)	
H15	0.9294	0.5841	0.5033	0.065*	
C16	0.8501 (3)	0.3510 (3)	0.6659 (2)	0.0603 (10)	
H16A	0.9244	0.3438	0.6930	0.090*	
H16B	0.8188	0.2917	0.6481	0.090*	
H16C	0.8199	0.3784	0.7096	0.090*	
C17	0.6908 (2)	0.87357 (19)	0.2698 (2)	0.0348 (7)	
H17	0.7554	0.8953	0.2669	0.042*	
C18	0.5341 (2)	0.8715 (2)	0.2899 (2)	0.0372 (7)	
C19	0.5603 (2)	0.7845 (2)	0.2655 (2)	0.0356 (7)	
C20	0.4904 (3)	0.7117 (2)	0.2553 (3)	0.0589 (10)	
H20	0.5074	0.6532	0.2392	0.071*	
C21	0.3955 (4)	0.7290 (3)	0.2696 (4)	0.0806 (15)	
H21	0.3471	0.6813	0.2628	0.097*	
C22	0.3697 (3)	0.8165 (3)	0.2944 (3)	0.0742 (13)	
H22	0.3048	0.8256	0.3044	0.089*	
C23	0.4375 (3)	0.8892 (3)	0.3041 (3)	0.0550 (10)	
H23	0.4198	0.9478	0.3196	0.066*	
C24	0.5534 (3)	0.7473 (2)	0.0435 (2)	0.0411 (8)	
H24	0.5471	0.7972	0.0797	0.049*	
C25	0.5272 (2)	0.6580 (2)	−0.0758 (2)	0.0368 (7)	
C26	0.4969 (3)	0.6177 (2)	−0.1616 (2)	0.0509 (9)	
H26	0.4460	0.6443	−0.2103	0.061*	
C27	0.5453 (4)	0.5368 (3)	−0.1715 (3)	0.0814 (16)	
H27	0.5275	0.5077	−0.2282	0.098*	
C28	0.6212 (4)	0.4971 (3)	−0.0973 (3)	0.0939 (19)	
H28	0.6522	0.4419	−0.1061	0.113*	
C29	0.6513 (3)	0.5373 (2)	−0.0118 (3)	0.0689 (13)	
H29	0.7010	0.5099	0.0371	0.083*	
C30	0.6042 (2)	0.6207 (2)	−0.0017 (2)	0.0381 (7)	
Co1	0.73145 (3)	0.68195 (2)	0.20032 (3)	0.02980 (12)	
N1	0.61978 (19)	0.67861 (16)	0.07391 (16)	0.0346 (6)	
N2	0.4965 (2)	0.73853 (18)	−0.04404 (17)	0.0387 (6)	
H2	0.4494	0.7762	−0.0747	0.046*	
N3	0.65969 (19)	0.78738 (15)	0.25232 (16)	0.0326 (5)	
N4	0.6196 (2)	0.92647 (16)	0.29238 (17)	0.0377 (6)	
H4A	0.6260	0.9841	0.3059	0.045*	
O1	0.85066 (16)	0.75360 (13)	0.17053 (14)	0.0347 (5)	
O2	0.85031 (16)	0.60520 (13)	0.14347 (14)	0.0359 (5)	
O3	0.70607 (16)	0.56763 (13)	0.27254 (13)	0.0340 (5)	
O4	0.84150 (16)	0.65163 (13)	0.34567 (13)	0.0368 (5)	

### Atomic displacement parameters (Å^2^)

**Table:**

	*U*^11^	*U*^22^	*U*^33^	*U*^12^	*U*^13^	*U*^23^
C1	0.0313 (15)	0.0250 (13)	0.0310 (14)	0.0020 (12)	−0.0005 (12)	0.0031 (12)
C2	0.0346 (16)	0.0307 (15)	0.0292 (14)	0.0034 (12)	0.0023 (12)	0.0058 (12)
C3	0.050 (2)	0.0316 (16)	0.055 (2)	−0.0030 (15)	0.0202 (17)	−0.0036 (15)
C4	0.054 (2)	0.0427 (19)	0.064 (2)	−0.0119 (17)	0.0201 (19)	0.0022 (17)
C5	0.0439 (19)	0.0492 (19)	0.0397 (18)	0.0022 (16)	0.0121 (15)	0.0085 (15)
C6	0.053 (2)	0.0419 (18)	0.046 (2)	0.0054 (16)	0.0173 (17)	0.0019 (15)
C7	0.0444 (19)	0.0323 (16)	0.0425 (18)	−0.0010 (14)	0.0103 (15)	0.0012 (14)
C8	0.064 (3)	0.071 (3)	0.066 (3)	−0.006 (2)	0.032 (2)	0.007 (2)
C9	0.0304 (15)	0.0238 (13)	0.0333 (15)	−0.0010 (12)	0.0050 (12)	−0.0068 (12)
C10	0.0366 (17)	0.0252 (14)	0.0302 (15)	0.0003 (12)	0.0047 (13)	−0.0019 (12)
C11	0.048 (2)	0.0497 (19)	0.0348 (17)	−0.0165 (16)	0.0026 (15)	−0.0002 (15)
C12	0.063 (2)	0.053 (2)	0.0426 (19)	−0.0238 (18)	0.0107 (18)	0.0033 (17)
C13	0.0453 (19)	0.0419 (17)	0.0364 (17)	0.0000 (15)	0.0112 (15)	0.0043 (14)
C14	0.059 (2)	0.055 (2)	0.0392 (19)	−0.0156 (19)	−0.0111 (17)	0.0093 (17)
C15	0.054 (2)	0.047 (2)	0.045 (2)	−0.0251 (17)	−0.0123 (17)	0.0124 (16)
C16	0.075 (3)	0.064 (2)	0.041 (2)	−0.006 (2)	0.0161 (19)	0.0134 (18)
C17	0.0373 (17)	0.0269 (14)	0.0374 (16)	−0.0011 (13)	0.0066 (14)	0.0012 (12)
C18	0.0403 (18)	0.0321 (16)	0.0371 (17)	0.0006 (13)	0.0080 (14)	−0.0054 (13)
C19	0.0363 (17)	0.0309 (15)	0.0372 (16)	−0.0009 (13)	0.0070 (14)	−0.0039 (13)
C20	0.060 (2)	0.0373 (18)	0.085 (3)	−0.0164 (17)	0.030 (2)	−0.0177 (19)
C21	0.062 (3)	0.062 (3)	0.132 (4)	−0.028 (2)	0.050 (3)	−0.028 (3)
C22	0.054 (3)	0.074 (3)	0.106 (4)	−0.012 (2)	0.041 (3)	−0.025 (3)
C23	0.047 (2)	0.050 (2)	0.067 (2)	0.0048 (17)	0.0159 (19)	−0.0165 (18)
C24	0.0419 (19)	0.0420 (17)	0.0359 (17)	0.0160 (15)	0.0057 (14)	0.0024 (14)
C25	0.0305 (16)	0.0295 (15)	0.0418 (17)	−0.0006 (12)	−0.0029 (14)	0.0042 (13)
C26	0.054 (2)	0.0337 (17)	0.0445 (19)	0.0019 (16)	−0.0171 (16)	0.0004 (15)
C27	0.110 (4)	0.041 (2)	0.057 (2)	0.020 (2)	−0.032 (2)	−0.0226 (19)
C28	0.120 (4)	0.043 (2)	0.073 (3)	0.042 (2)	−0.042 (3)	−0.027 (2)
C29	0.079 (3)	0.0350 (18)	0.060 (2)	0.0212 (19)	−0.031 (2)	−0.0129 (17)
C30	0.0364 (17)	0.0276 (14)	0.0386 (17)	−0.0007 (13)	−0.0071 (14)	−0.0009 (13)
Co1	0.0307 (2)	0.02232 (18)	0.0311 (2)	0.00253 (17)	0.00082 (16)	−0.00087 (16)
N1	0.0317 (13)	0.0303 (12)	0.0337 (13)	0.0065 (11)	−0.0030 (11)	0.0002 (11)
N2	0.0334 (15)	0.0429 (15)	0.0342 (14)	0.0135 (12)	0.0008 (11)	0.0033 (12)
N3	0.0365 (14)	0.0233 (11)	0.0354 (13)	−0.0015 (10)	0.0065 (11)	−0.0023 (10)
N4	0.0419 (15)	0.0210 (12)	0.0471 (16)	0.0001 (11)	0.0083 (12)	−0.0047 (11)
O1	0.0374 (12)	0.0251 (10)	0.0383 (11)	0.0017 (9)	0.0062 (9)	0.0001 (9)
O2	0.0367 (12)	0.0245 (10)	0.0426 (12)	0.0000 (9)	0.0054 (10)	0.0026 (9)
O3	0.0352 (12)	0.0277 (10)	0.0308 (11)	−0.0015 (9)	−0.0033 (9)	−0.0016 (8)
O4	0.0385 (12)	0.0314 (10)	0.0323 (11)	−0.0073 (9)	−0.0027 (9)	0.0016 (9)

### Geometric parameters (Å, °)

**Table:**

C1—O2	1.265 (3)	C17—H17	0.9300
C1—O1	1.270 (3)	C18—N4	1.382 (4)
C1—C2	1.482 (4)	C18—C19	1.390 (4)
C2—C7	1.391 (4)	C18—C23	1.390 (5)
C2—C3	1.395 (4)	C19—C20	1.387 (4)
C3—C4	1.381 (5)	C19—N3	1.397 (4)
C3—H3	0.9300	C20—C21	1.367 (5)
C4—C5	1.385 (5)	C20—H20	0.9300
C4—H4	0.9300	C21—C22	1.397 (6)
C5—C6	1.381 (5)	C21—H21	0.9300
C5—C8	1.505 (5)	C22—C23	1.369 (5)
C6—C7	1.376 (4)	C22—H22	0.9300
C6—H6	0.9300	C23—H23	0.9300
C7—H7	0.9300	C24—N1	1.323 (4)
C8—H8A	0.9600	C24—N2	1.335 (4)
C8—H8B	0.9600	C24—H24	0.9300
C8—H8C	0.9600	C25—N2	1.372 (4)
C9—O4	1.262 (3)	C25—C26	1.383 (4)
C9—O3	1.273 (3)	C25—C30	1.395 (4)
C9—C10	1.494 (4)	C26—C27	1.369 (5)
C10—C11	1.373 (4)	C26—H26	0.9300
C10—C15	1.380 (4)	C27—C28	1.402 (5)
C11—C12	1.389 (5)	C27—H27	0.9300
C11—H11	0.9300	C28—C29	1.378 (5)
C12—C13	1.384 (5)	C28—H28	0.9300
C12—H12	0.9300	C29—C30	1.391 (4)
C13—C14	1.359 (5)	C29—H29	0.9300
C13—C16	1.511 (4)	C30—N1	1.394 (4)
C14—C15	1.377 (5)	Co1—O1	2.057 (2)
C14—H14	0.9300	Co1—N1	2.064 (2)
C15—H15	0.9300	Co1—O3	2.074 (2)
C16—H16A	0.9600	Co1—N3	2.081 (2)
C16—H16B	0.9600	Co1—O2	2.303 (2)
C16—H16C	0.9600	Co1—O4	2.316 (2)
C17—N3	1.320 (4)	N2—H2	0.8600
C17—N4	1.341 (4)	N4—H4A	0.8600
			
O2—C1—O1	119.7 (3)	C21—C20—H20	121.2
O2—C1—C2	120.6 (3)	C19—C20—H20	121.2
O1—C1—C2	119.7 (2)	C20—C21—C22	121.5 (4)
C7—C2—C3	118.3 (3)	C20—C21—H21	119.2
C7—C2—C1	120.8 (3)	C22—C21—H21	119.2
C3—C2—C1	120.8 (3)	C23—C22—C21	121.6 (4)
C4—C3—C2	120.0 (3)	C23—C22—H22	119.2
C4—C3—H3	120.0	C21—C22—H22	119.2
C2—C3—H3	120.0	C22—C23—C18	116.9 (3)
C3—C4—C5	121.7 (3)	C22—C23—H23	121.6
C3—C4—H4	119.2	C18—C23—H23	121.6
C5—C4—H4	119.2	N1—C24—N2	113.9 (3)
C6—C5—C4	117.9 (3)	N1—C24—H24	123.0
C6—C5—C8	121.3 (3)	N2—C24—H24	123.0
C4—C5—C8	120.8 (3)	N2—C25—C26	131.3 (3)
C7—C6—C5	121.4 (3)	N2—C25—C30	105.6 (3)
C7—C6—H6	119.3	C26—C25—C30	123.1 (3)
C5—C6—H6	119.3	C27—C26—C25	116.8 (3)
C6—C7—C2	120.7 (3)	C27—C26—H26	121.6
C6—C7—H7	119.6	C25—C26—H26	121.6
C2—C7—H7	119.6	C26—C27—C28	121.0 (3)
C5—C8—H8A	109.5	C26—C27—H27	119.5
C5—C8—H8B	109.5	C28—C27—H27	119.5
H8A—C8—H8B	109.5	C29—C28—C27	122.2 (4)
C5—C8—H8C	109.5	C29—C28—H28	118.9
H8A—C8—H8C	109.5	C27—C28—H28	118.9
H8B—C8—H8C	109.5	C28—C29—C30	117.2 (3)
O4—C9—O3	119.8 (3)	C28—C29—H29	121.4
O4—C9—C10	120.6 (3)	C30—C29—H29	121.4
O3—C9—C10	119.7 (2)	C29—C30—C25	119.8 (3)
C11—C10—C15	117.8 (3)	C29—C30—N1	130.8 (3)
C11—C10—C9	121.6 (3)	C25—C30—N1	109.4 (3)
C15—C10—C9	120.6 (3)	O1—Co1—N1	101.36 (9)
C10—C11—C12	120.7 (3)	O1—Co1—O3	141.11 (8)
C10—C11—H11	119.6	N1—Co1—O3	107.06 (9)
C12—C11—H11	119.6	O1—Co1—N3	100.55 (9)
C13—C12—C11	121.2 (3)	N1—Co1—N3	95.05 (10)
C13—C12—H12	119.4	O3—Co1—N3	102.76 (9)
C11—C12—H12	119.4	O1—Co1—O2	60.06 (7)
C14—C13—C12	117.4 (3)	N1—Co1—O2	91.33 (9)
C14—C13—C16	120.8 (3)	O3—Co1—O2	92.89 (7)
C12—C13—C16	121.7 (3)	N3—Co1—O2	160.49 (8)
C13—C14—C15	121.9 (3)	O1—Co1—O4	89.70 (8)
C13—C14—H14	119.0	N1—Co1—O4	166.53 (9)
C15—C14—H14	119.0	O3—Co1—O4	59.64 (7)
C14—C15—C10	121.0 (3)	N3—Co1—O4	90.39 (9)
C14—C15—H15	119.5	O2—Co1—O4	87.53 (7)
C10—C15—H15	119.5	C24—N1—C30	104.0 (2)
C13—C16—H16A	109.5	C24—N1—Co1	122.4 (2)
C13—C16—H16B	109.5	C30—N1—Co1	132.88 (19)
H16A—C16—H16B	109.5	C24—N2—C25	107.1 (2)
C13—C16—H16C	109.5	C24—N2—H2	126.5
H16A—C16—H16C	109.5	C25—N2—H2	126.5
H16B—C16—H16C	109.5	C17—N3—C19	104.7 (2)
N3—C17—N4	113.0 (3)	C17—N3—Co1	128.5 (2)
N3—C17—H17	123.5	C19—N3—Co1	126.41 (19)
N4—C17—H17	123.5	C17—N4—C18	107.6 (2)
N4—C18—C19	105.2 (3)	C17—N4—H4A	126.2
N4—C18—C23	133.0 (3)	C18—N4—H4A	126.2
C19—C18—C23	121.8 (3)	C1—O1—Co1	95.61 (17)
C18—C19—C20	120.6 (3)	C1—O2—Co1	84.59 (16)
C18—C19—N3	109.5 (3)	C9—O3—Co1	95.55 (17)
C20—C19—N3	129.8 (3)	C9—O4—Co1	84.87 (16)
C21—C20—C19	117.6 (3)		

### Hydrogen-bond geometry (Å, °)

**Table:**

*D*—H···*A*	*D*—H	H···*A*	*D*···*A*	*D*—H···*A*
N2—H2···O4^i^	0.86	1.90	2.757 (3)	173
N4—H4A···O2^ii^	0.86	1.91	2.760 (3)	170

Symmetry codes: (i) *x*−1/2, −*y*+3/2, *z*−1/2; (ii) −*x*+3/2, *y*+1/2, −*z*+1/2.
